# Needle as a Foreign Body in the Kidney of an Infant: A Case Report

**DOI:** 10.7759/cureus.81685

**Published:** 2025-04-04

**Authors:** VK Gopi, Dilsa Savio, Ramdas E.K

**Affiliations:** 1 Department of Pediatric Surgery, Baby Memorial Hospital, Kozhikode, IND; 2 Department of Anesthesiology, Baby Memorial Hospital, Kozhikode, IND

**Keywords:** broken needle, laparoscopic retrieval, renal foreign body, unnoticed needle injury, unusual foreign body

## Abstract

Foreign bodies in the kidneys are extremely rare, particularly in pediatric patients. We report a unique case of a nine-month-old male infant who was incidentally found to have a needle in his left kidney during evaluation for a lower respiratory tract infection. The foreign body was initially identified on chest X-ray and confirmed by a computed tomography scan. The infant had a history of transient swelling in the left lumbar region during the neonatal period, which resolved spontaneously, suggesting a possible entry point. Despite being asymptomatic, surgical intervention was deemed necessary due to the metallic nature of the foreign body and its potential for complications. A laparoscopic approach was successfully used to remove the 3 cm needle, which was identified as a broken hypodermic injection needle. The kidney was preserved, and no postoperative complications were observed. The infant's recovery was uneventful, with normal renal function and imaging during follow-up. This case highlights the rarity of renal foreign bodies in infants, the effectiveness of laparoscopic surgery for their removal, and the importance of early recognition and timely intervention to prevent long-term complications. The report also discusses potential risks associated with outdated practices, such as the broken needle technique, which involves intentionally breaking a hypodermic needle for collecting blood samples from neonates and infants, and emphasizes the need for improved safety protocols and awareness in pediatric procedures.

## Introduction

Foreign bodies in the urinary tract, especially in the kidneys, are quite rare and often present unique clinical challenges. In most cases, they result from endourological procedures, trauma, intentional insertion, sometimes seen in older children or adults or even the unexpected translocation of ingested objects into the kidneys [1-3]. While foreign bodies in the urinary system are uncommon, they tend to be more frequently encountered in adults or older children, particularly those with a history of trauma or self-insertion [4,5]. However, finding a foreign body lodged in the kidney of an infant is exceptionally rare, with only a few such cases documented in medical literature [6].

One of the biggest challenges in these cases is that foreign bodies in the kidneys often go unnoticed for a long time. Many patients don’t show any obvious symptoms, or they might only experience vague signs like fever, abdominal pain, or hematuria [7,8]. In fact, many of these objects are discovered purely by accident during imaging done for an entirely different reason [9]. This makes their diagnosis tricky and their management even more complex, especially in infants, where factors like age, risk of complications, and the safest method of removal must all be carefully considered [4].

Here, we present a rare and unusual case of a nine-month-old infant who was found to have a needle embedded in the kidney. What makes this case even more remarkable is that the needle was successfully removed using a laparoscopic approach, a technique that is not commonly performed in such young children. While laparoscopic retrieval of foreign bodies from the kidney has been reported in adults [9], its use in infants under one year of age is almost unheard of. This case highlights not only the rarity of the condition but also how minimally invasive techniques can offer safer and more effective treatment options, even in the most delicate of patients.

## Case presentation

**Figure 1:**
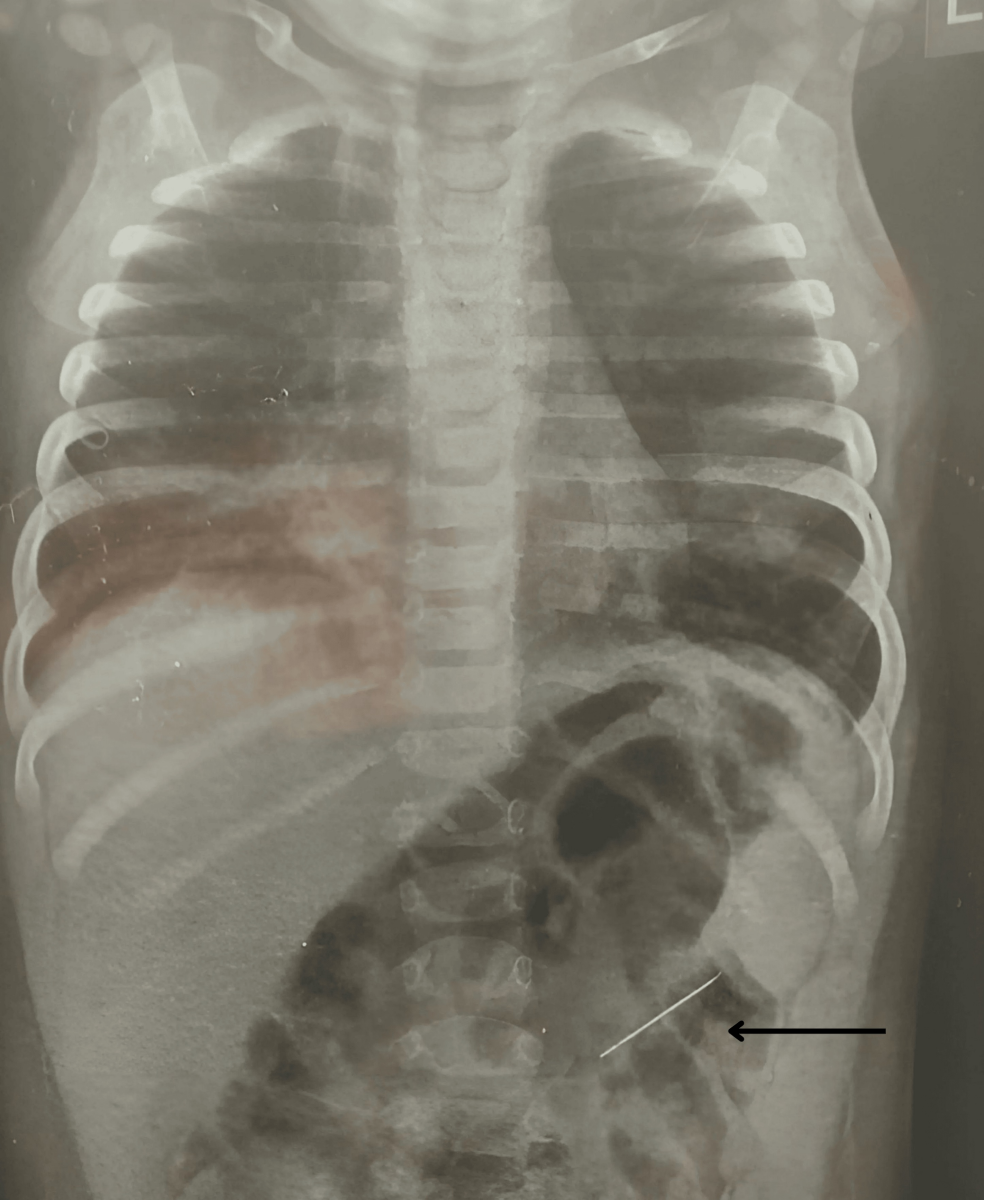
X-ray showing a radio-opaque linear foreign body in the left lumbar region.

**Figure 2:**
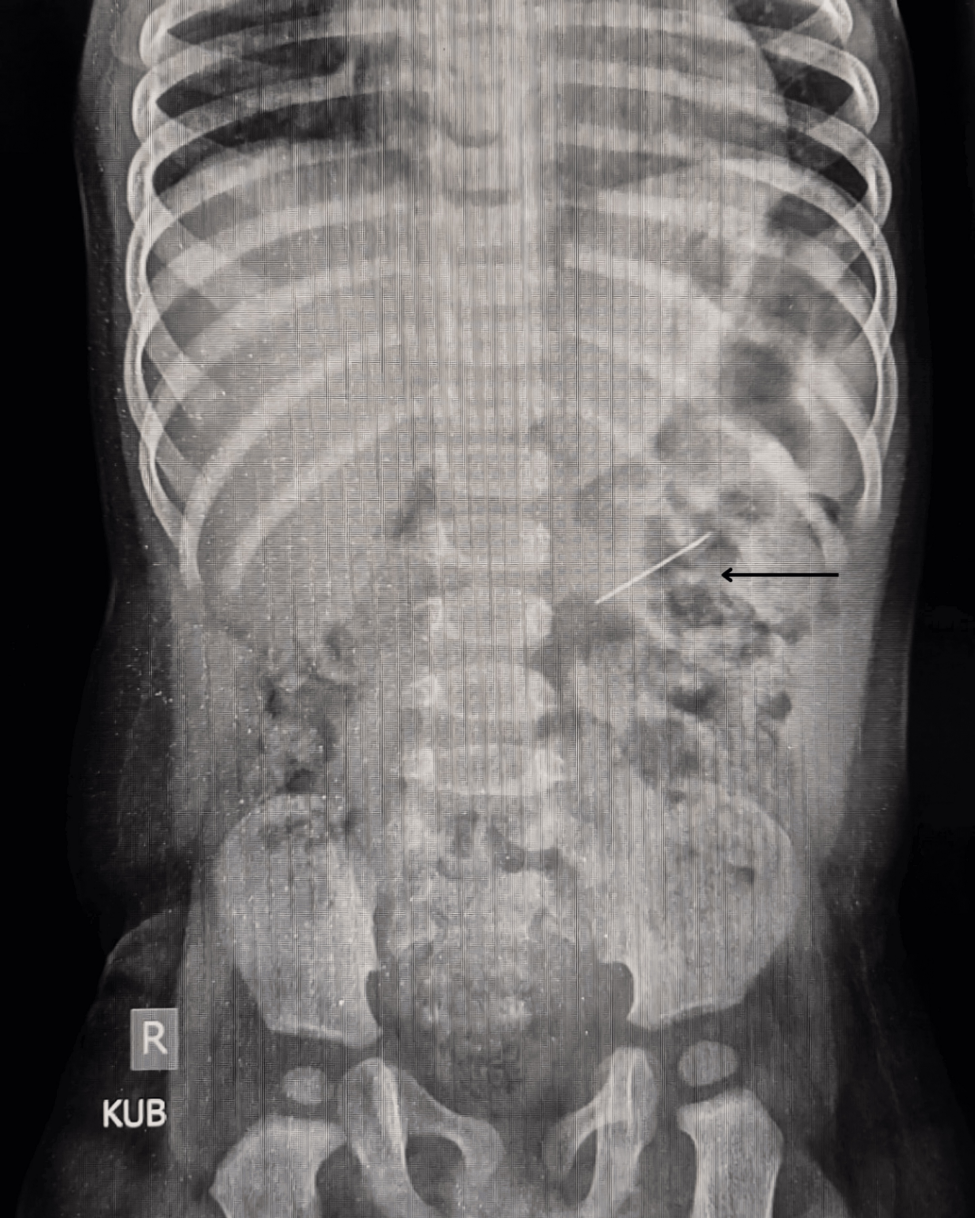
X-ray KUB showing a linear radio opaque foreign body in the left lumbar region. KUB: Kidney and urinary bladder

**Figure 3:**
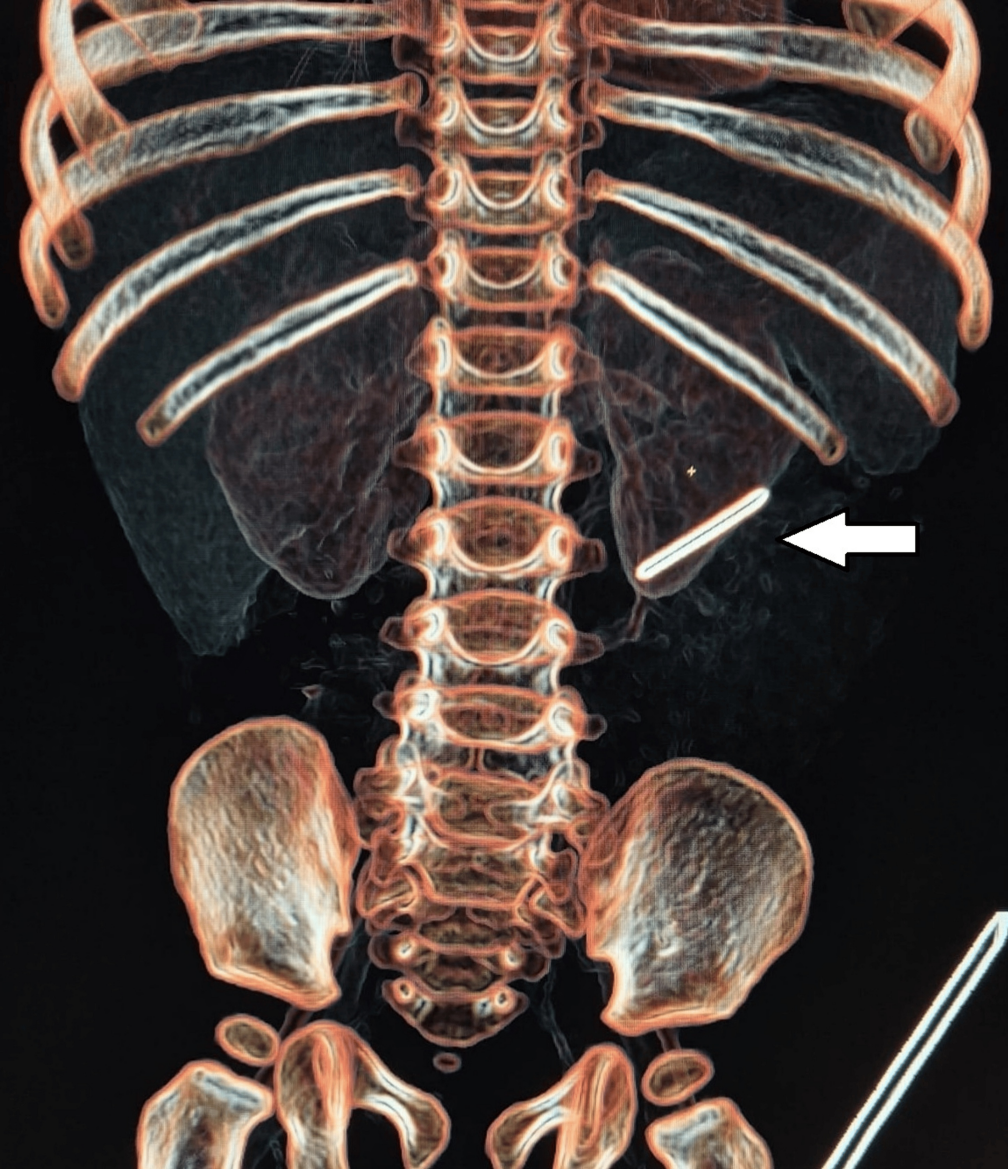
3D reconstructed computed tomography (CT) image showing a linear foreign body in the lower pole of the left kidney.

A nine-month-old male infant was referred to our facility following the incidental discovery of a metallic foreign body on a chest X-ray taken during evaluation for a lower respiratory tract infection (LRTI) (Figure 1). An X-ray kidney and urinary bladder (KUB) showed a metallic object resembling a needle in the left lumbar region (Figure 2). Further evaluation with the CT scan showed a linear hyperdense focus (foreign body) in relation to the lower pole of the left kidney (Figure 3 and Video 1).

**Video 1:** CT scan showing a linear foreign body in the left kidney lower pole.

**Figure 4:**
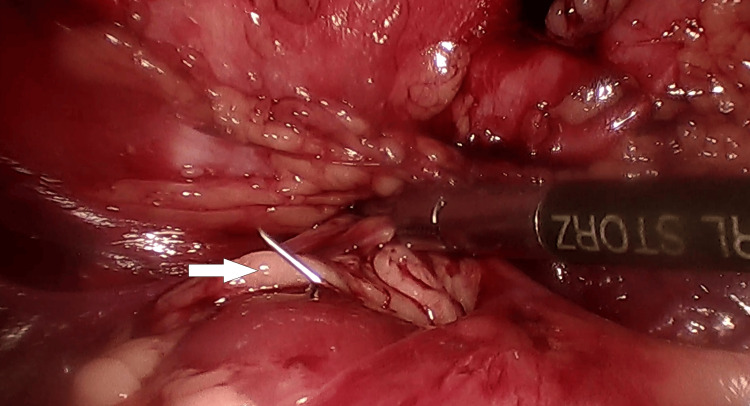
Figure showing the needle emerging from the anterior surface of the left kidney.

The infant had a brief admission to the neonatal ICU, and a few days later, swelling over the left loin was noticed, which spontaneously resolved within a few days. Since then, the patient has remained asymptomatic. The infant demonstrated normal growth and developmental milestones appropriate for age. The infant was asymptomatic, with stable vital signs and no clinical abnormalities. All blood investigations, including complete blood counts and renal function tests, were within normal limits. Considering the metallic nature of the foreign body and its potential complications, surgical intervention was deemed necessary. The laparoscopic technique was selected because of its safety profile and minimally invasive nature [3,9]. A 3 cm-long needle was found poking from the left renal parenchyma during the surgery (Figure 4). The needle was carefully removed without damaging the surrounding renal tissue. Upon closer examination, the foreign body was identified as a broken hypodermic injection needle. The kidney was preserved, and hemostasis was successfully achieved (Video 2).

**Video 2:** Laparoscopic retrieval of the broken needle.

The infant’s postoperative recovery was uneventful. He was discharged on the third postoperative day and remained asymptomatic during follow-up. Imaging confirmed the normal appearing left kidney, and renal function tests were within normal limits.

## Discussion

Foreign bodies in the kidneys are extremely rare, especially in the pediatric population. Most reported cases in the literature involve adults, often associated with intentional self-insertion, traumatic injuries, or iatrogenic causes during endourological procedures or translocation of ingested foreign bodies [1,2,4,5]. In children, these occurrences are extremely uncommon, with only a handful of documented cases, and the presence of a foreign body in an infant's kidney is even rarer.

The history of swelling in the left lumbar region during the neonatal period, which resolved spontaneously, suggests a possible entry point. It is plausible that the needle was inadvertently introduced during the neonatal period through an unnoticed injury or accidental puncture. The soft and pliable nature of infant tissues may have facilitated the migration of the needle over time into the kidney, aided by bodily movements or the forces exerted by surrounding tissues [4,6]. Migration of foreign bodies into internal organs has been documented in other anatomical locations, where foreign bodies were found far from their initial points of entry [7,8].

Clinically, the child was asymptomatic, highlighting the silent nature of some foreign bodies in the urinary tract. While adults more commonly present with symptoms such as hematuria, flank pain, infection, or urinary obstruction, pediatric patients may remain asymptomatic for prolonged periods, as seen in this case. However, symptoms can develop if complications such as infection, obstruction, or tissue damage occur. The needle was incidentally discovered during imaging for an LRTI, highlighting the crucial role of imaging studies in detecting foreign bodies that might otherwise go unnoticed. X-rays and subsequent imaging modalities are essential for localizing metallic foreign bodies and evaluating potential complications, such as organ damage, abscess formation, or obstruction [1,4].

The choice of management for renal foreign bodies depends on factors such as size, location, associated symptoms, and the potential risk of complications. In this case, the asymptomatic nature of the foreign body allowed for careful planning of the minimally invasive surgical approach, successfully avoiding both laparotomy and even nephrectomy. Minimal access surgical techniques, particularly laparoscopy, have become the standard of care in pediatric urology due to their numerous advantages, including reduced postoperative pain, faster recovery, shorter hospital stays, and better cosmetic outcomes [1,9]. The successful laparoscopic retrieval of the needle in this case highlights the efficacy and safety of this approach, especially in delicate cases involving young infants. The preservation of renal function, the avoidance of laparotomy and nephrectomy, and the uneventful postoperative recovery further underscore the importance of minimal access surgery in managing such rare cases.

One of the significant risks of retained foreign bodies in the kidney is the development of long-term complications. These may include infections, abscess formation, chronic inflammation, obstruction of the urinary tract, bleeding, or even loss of renal function. Early recognition and timely removal of the foreign body are essential to prevent these outcomes [1,2]. Despite the asymptomatic presentation in this case, the decision to remove the needle was justified due to its potential to cause future complications. The absence of postoperative complications and the preservation of renal function reaffirm the importance of proactive surgical intervention.

This case underscores the need for maintaining a high index of suspicion for foreign bodies in unusual locations, particularly in pediatric patients with incomplete or absent history. The presence of a foreign body in the kidney of a young child raises important considerations regarding its possible entry mechanism. In this instance, the transient swelling observed during the neonatal period suggests a potential unnoticed penetrating injury, likely from an iatrogenic cause such as an inadvertent needle puncture during medical procedures. The delicate and pliable nature of infant tissues may have facilitated the gradual migration of the foreign body into the renal parenchyma over time.

The broken needle technique [10], once common in the UK but still practiced in parts of India, may have played a role in this case. This method, which involves deliberately breaking a hypodermic needle to aid blood collection from small veins in neonates, poses serious risks. These include accidental punctures and misplacement, potentially resulting in internal migration of the needle. In this case, a broken needle used for Venepuncture was likely misplaced, resulting in an unrecognized injury. The combination of a broken needle and improper disposal may have contributed to the accidental puncture. Without the awareness of the caregiver or medical professional, the needle appears to have entered through the lumbar region and subsequently migrated into the renal parenchyma. This case highlights the potential risks associated with the use of broken needles and underscores the importance of proper disposal practices to prevent such incidents.

To mitigate the risk of such occurrences, healthcare providers must adhere strictly to safety protocols, particularly when handling sharp instruments like needles and catheters during neonatal and pediatric procedures. Proper disposal of medical equipment, especially broken needles, is essential to prevent accidental injuries [11]. Also, quality reports should be generated to ensure detailed case analysis, facilitate learning, and improve patient safety in similar scenarios.

Improved documentation and clear communication of medical interventions can help identify potential iatrogenic injuries early. Additionally, raising awareness among healthcare providers about the risks of outdated practices, such as the broken needle technique, is crucial. Emphasizing safer methods for blood sampling, such as using smaller needles or alternative devices, should be practiced to minimize risks.

Parental education on the potential hazards of foreign bodies including accidental ingestion or injury can further enhance prevention strategies. Ensuring proper technique, post-procedure inspection, and heightened vigilance during invasive procedures are key to reducing the risk of foreign body retention and improving patient safety.

## Conclusions

This report details an unusual case of a nine-month-old infant with a broken needle foreign body in the kidney, which was detected incidentally while imaging for an unrelated condition. Its laparoscopic retrieval also demonstrates the successful use of minimally invasive surgery in children, which provides a safe and effective management solution. Pediatric foreign bodies should be in the differential diagnosis of atypical clinical signs and imaging results. These cases require a multidisciplinary collaboration including pediatricians, surgeons, anesthesiologists, and radiologists to come to an accurate and timely diagnosis, effective management, and better outcomes.

This report aims to raise awareness and provide guidance for clinicians managing such scenarios by adding to the limited literature available on pediatric renal foreign bodies. It also illustrates the need for increased caution during neonatal interventions as well as the development of better blood collection methods and disposal protocols.
